# Supplementary material for: Exploring the phase stability in interpenetrated diamondoid covalent organic frameworks
Source: Commun Chem. 2023 Jan 6;6:5. doi: 10.1038/s42004-022-00808-y (PMC9822923; doi:10.1038/s42004-022-00808-y)
Supplement: Supplementary file 2 — Supplementary Information [file 42004_2022_808_MOESM2_ESM.pdf]

Supporting Information

for

Exploring the phase stability in interpenetrated diamondoid covalent organic frameworks

Sander Borgmans,[†] Sven M. J. Rogge,[*,†] Juul S. De Vos,[†] Pascal Van Der Voort,[‡] and Veronique Van Speybroeck[*,†]

Center for Molecular Modeling (CMM), Ghent University, Technologiepark-Zwijnaarde 46, 9052 Zwijnaarde, Belgium, and Center for Ordered Materials, Organometallics and Catalysis (COMOC), Department of Inorganic and Physical Chemistry, Ghent University, Krijgslaan 281 (S3), 9000 Gent, Belgium

Supplementary Note 1 — *In silico* generation of interpenetrated structures — S-3

Supplementary Note 1.1 Structure generation S-3

Supplementary Note 1.2 Interpenetrated dia nets S-4

Supplementary Note 2 — Experimental reference data — S-6

Supplementary Note 3 — Correlation between collective variables and system properties — S-8

Supplementary Note 4 — Iterative WHAM algorithm — S-10

Supplementary Note 5 — Free energy landscapes — S-13

Supplementary Note 5.1 Empty framework S-13

Supplementary Note 5.2 Water-filled framework S-21

Supplementary Note 5.3 Derivation of transition barriers S-21

Supplementary Note 6 — From *ab initio* cluster structures to periodic force field structures — S-23

Supplementary Note 6.1 Generation of cluster models S-23
Supplementary Note 6.2 Validation procedure S-25
Supplementary Note 6.3 Combining cluster force fields S-27
Supplementary Note 6.4 Additional force field terms S-27
Supplementary Note 6.5 Modelling water-framework interactions S-30

Supplementary Note 1 *In silico* generation of interpenetrated structures

As elaborated in the main text (and depicted in Supplementary Figure 17), four different materials are considered, creating a versatile set of interpenetrated covalent organic frameworks (COFs). COF-300 and COF-320[1] are formed by tetratopic tetra-(4-anilyl)methane, and either ditopic terephthalaldehyde (COF-300) or 4-(4-formylphenyl)benzaldehyde (COF-320) building blocks. Notably, they differ only in the length of their linker, with COF-300 incorporating the shorter linker. In contrast, NPN-1[2] and NPN-3,[2] formed through the selfcondensation of tetrakis(4-nitrosophenyl)methane or 1,3,5,7-tetrakis(4-nitrosophenyl)adamantane, respectively, are fundamentally different with a much shorter and more rigid linker, and, for NPN-3, a more rigid tetratopic building block with an adamantane cage instead of a tetraphenylmethane moiety.

Supplementary Note 1.1 Structure generation

All initial materials are generated *in silico* using our in-house structure assembly software, which is based on a top-down approach, starting from the building blocks of the material and the underlying topology. The building blocks of the four COFs are derived from the cluster models, which are visualised in Supplementary Figure 17. After a force field optimization, appropriate connection points are defined as the points where the building block can link with other building blocks. There are as many connection points as the coordination number of the building block designates. For the building blocks that constitute the imine-linked COFs (COF-300 and COF-320), they are chosen in the centre of the C-N imine bond, whereas for the building blocks forming the azodioxy-linked COFs (NPN-1 and NPN-3), they are positioned in the centre of the N-N azodioxy bond. The initial geometries of the building blocks are obtained from the force field optimised clusters, from which the termination, *i.e.* all atoms that are beyond the connection points, are omitted. All topologies are derived from the non-interpenetrated **dia** net. A mathematical description of the interpenetrated topologies is given in Section Supplementary Note 1.2. As mentioned in the main text, the degree of interpenetration is varied from onefold (non-interpenetrated) to eightfold (or elevenfold for COF-320, as the linker in this COF is especially longer than the other linkers and steric repulsion only becomes important at higher degrees of interpenetration).

Once the building blocks and the topology are selected, its nodes are decorated in a three step procedure. Firstly, the unit cell of the topology is rescaled isotropically as such that the building

blocks can be correctly positioned on its nodes. Secondly, the configurations of each building block in which the connection points are nicely oriented towards the neighbouring topological nodes are selected. As the symmetry of the atomic representation of the building blocks is lower than the symmetry of its connection points, multiple configurations result in the same alignment within the topology. For example, a rotation of the ditopic building blocks around the axis formed by its two connection points, keeps their positions fixed, while the atomic positions are changed. In the last step, a final configuration is selected from the remaining ones based on an energetical descriptor that defines the energy penalty that is introduced by inserting the building block in this specific configuration. Once all topological nodes are decorated with the appropriate building blocks, the final structure is relaxed with the periodic force fields as derived in Section Supplementary Note 6.

Supplementary Note 1.2 Interpenetrated dia nets

As explained in the previous section, the topological net is required as input for the structure assembly software. The non-interpenetrated diamondoid topology can be represented by four vertices in an orthogonal unit cell with the length of the **c** vector equal to 2.3094 Å and the length of the **a** and **b** vector being 1.6330 Å ($= \frac{\sqrt{2}}{2} \|\mathbf{c}\|$). These values are normalised such that the distance between two connected vertices is equal to one. In this unit cell, the coordinates of the four vertices are:

$$\mathbf{r}_1 = 0\mathbf{a} + \frac{1}{4}\mathbf{b} + \frac{5}{8}\mathbf{c} \tag{S1.1}$$

$$\mathbf{r}_2 = 0\mathbf{a} + \frac{3}{4}\mathbf{b} + \frac{3}{8}\mathbf{c} \tag{S1.2}$$

$$\mathbf{r}_3 = \frac{1}{2}\mathbf{a} + \frac{1}{4}\mathbf{b} + \frac{7}{8}\mathbf{c} \tag{S1.3}$$

$$\mathbf{r}_4 = \frac{1}{2}\mathbf{a} + \frac{3}{4}\mathbf{b} + \frac{1}{8}\mathbf{c} \tag{S1.4}$$

As the individual nets in an interpenetrated diamondoid topology can be constructed by translating along the twofold rotation axis (here the **c** axis), a straightforward approach to construct these topologies is by introducing four additional vertices in the unit cell that are translated from the original ones for each net. In general, to construct the k-th net in the n-fold interpenetrated net, each original node $\mathbf{r}_i$ should be translated with the vector $k\mathbf{c}/n$:

$$\mathbf{r}_i^{(k,n)} = \mathbf{r}_i + k\frac{\mathbf{c}}{n} \tag{S1.5}$$

However, applying this approach to construct the interpenetrated **dia** topologies, results in a non-consistent number of building blocks in the unit cell, that grows linearly, such that the results can not immediately be compared between the different degrees of interpenetration. A more elegant approach is to exploit the symmetry of the topology and the interpenetration axis, by introducing a new unit cell with the same **a** and **b** axis, but the scaling the original **c** axis by a factor $1/n$: $\mathbf{c}' = \mathbf{c}/n$. Equation S1.5 can now be rewritten as

$$\mathbf{r}_i^{(k,n)} = \mathbf{r}_i + k\mathbf{c}' \tag{S1.6}$$

Using this new unit cell, the individual nets now emerge as the periodic images of the original vertices $\mathbf{r}_i$ and do not have to be constructed explicitly. Furthermore, a consistent number of four vertices and eight edges are present in the unit cell, allowing for a direct comparison between the different degrees of interpenetration.

Supplementary Note 2 Experimental reference data

COF-300 is the archetypal interpenetrated **dia** covalent organic framework (COF), and has been extensively characterised, at varying conditions, with various techniques. This is captured in Table 1, where, together with other **dia** COFs, the reported degree of interpenetration and the respective 1D channel sizes have been provided as a reference for our free energy calculations. As discussed in the main text, four different materials were considered, creating a versatile set of interpenetrated COFs, namely, COF-300, COF-320, NPN-1, and NPN-3, for which the reported data has been summarised in Table 1. From this table, it is clear that while the **dia** topology potentially allows for different phases, flexibility does not necessarily come to expression in each of the materials, and occurs mostly in the presence of guest molecules.

Evidently, this potential for flexibility is correlated to the structural decoration for each material. Although COF-300 and COF-320 are extremely similar, no rectangular-shaped pore has yet been reported for COF-300, whereas it does exist for COF-320. Moreover, only a single phase is observed for the more rigid NPN-COFs. Furthermore, there are conflicting reports on the equilibrium channel size of COF-300(7). This could likely be attributed to the varying sample preparation, which can give rise to different crystal sizes, guest concentrations, among others.

Supplementary Table 1: Experimentally reported phases of COF-300, COF-320, NPN-1 and NPN-3, with their respective degree of interpenetration (between brackets), synthesis conditions, and channel shape. The corresponding values of the proposed collective variables, as defined in Figure 3 in the main text, are also reported. The channel shapes are referred to using the following symbols: ▭ = rectangular-shaped; ▫ = small square-shaped; □ = large square-shaped. When the phase is obtained through introducing guest molecules, the symbol is filled, and the guest molecule is specified. The following abbreviations are used in the table below: (PXRD) powder X-ray diffraction; (SXRD) single-crystal X-ray diffraction; (RED) rotation electron diffraction; (EDT) electron diffraction tomography; (atm) atmospheric conditions; (THF) tetrahydrofuran; (PMMA) poly(methyl methacrylate).

			conditions of the measurement				
material	*observed pore shape*	*guest molecules*	$CV_1 \times CV_2$ [Å×Å]	*method*	T	*synthetic procedure*	*REF*
COF-300(5)	□		14.1 × 14.1	PXRD	atm	solvothermal	[3]
COF-300(7)	▪	H_2O	9.8 × 9.8	SXRD	100 K	solvothermal	[4]
COF-300(7)	▪	H_2O	9.8 × 9.8	PXRD	atm	ventilation-vial	[5]
COF-300(7)	▪	H_2O	9.9 × 9.9	EDT	77 K	ventilation-vial	[6]
COF-300(7)	▫		10.2 × 10.2	PXRD	atm	ventilation-vial	[5]
COF-300(7)	▫		10.2 × 10.2	EDT	77 K	ventilation-vial	[6]
COF-300(7)	▫		10.2 × 10.2	RED/PXRD	93 K/298 K	solvothermal	[7]
COF-300(7)	□		13.1 × 13.1	SXRD	100 K	solvothermal	[4]
COF-300(7)	■	PMMA	13.6 × 13.6	EDT	77 K	ventilation-vial	[6]
COF-300(7)	■	THF	13.7 × 13.7	PXRD	atm	ventilation-vial	[5]
COF-320(9)	▭		17.7 × 12.5	RED	89 K	solvothermal	[1]
COF-320(9)	□		14.8 × 14.8	RED/PXRD	298 K/150 K	solvothermal	[1]
NPN-1(4)	▭		10.3 × 8.3	SXRD/PXRD	100 K/298 K	solvothermal	[2]
NPN-3(6)	□		11.2 × 11.2	SXRD/PXRD	100 K/298 K	solvothermal	[2]

Supplementary Note 3 Correlation between collective variables and system properties

Although the collective variable couple (CV_1,CV_2)is clearly defined, and easily related to the shape of the channel, its relation to cell parameters that can be experimentally measured is less obvious. To accommodate for this, Supplementary Figures 1 and 2 provide illustrative pictures relating the collective variables for the materials of Table 1 to the cell parameters ($a, b, c, \alpha, \beta, \gamma$) and the accessible pore volume (as calculated by Zeo++).[8,9] These properties are calculated as averages during individual trajectories of enhanced sampling simulations on a coarse grid of the collective variables to allow for a straightforward comparison to the free energy landscapes.

Notably, the asymmetry in the collective variables is not reflected in the cell parameters. This follows from the fact that within one unit cell four channels are captured, which cancel each other out, as illustrated in Supplementary Figure 1. Evidently, the pore volume is strongly correlated with channel size changes, as defined in the main text. However, shape variations also appear to have an indirect effect on the pore volume. This can be attributed to the changes in the elongation of the diamondoid cage, *i.e.* to variations of the c parameter. As already discussed in the main text, size decreases effectively correspond to an elongation of the diamondoid cage. However, when a shape change occurs, the nature of the steric interactions between the tetratopic building units changes. This results in a smaller elongation of the diamondoid cage for the same size, lowering the pore volume.

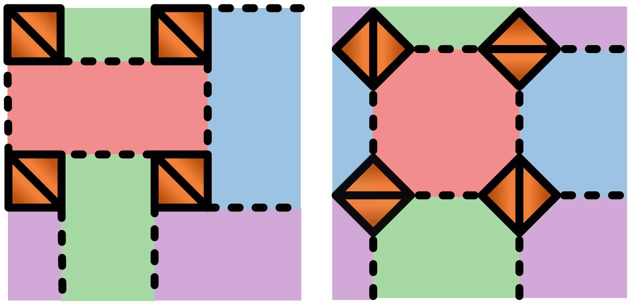

Supplementary Figure 1: Unit cell depiction for a system with squared and rectangular channels visualising the cancellation of the asymmetry of the channel shapes within a single unit cell. Each colour corresponds to a distinct channel, taking periodic boundary conditions into account.

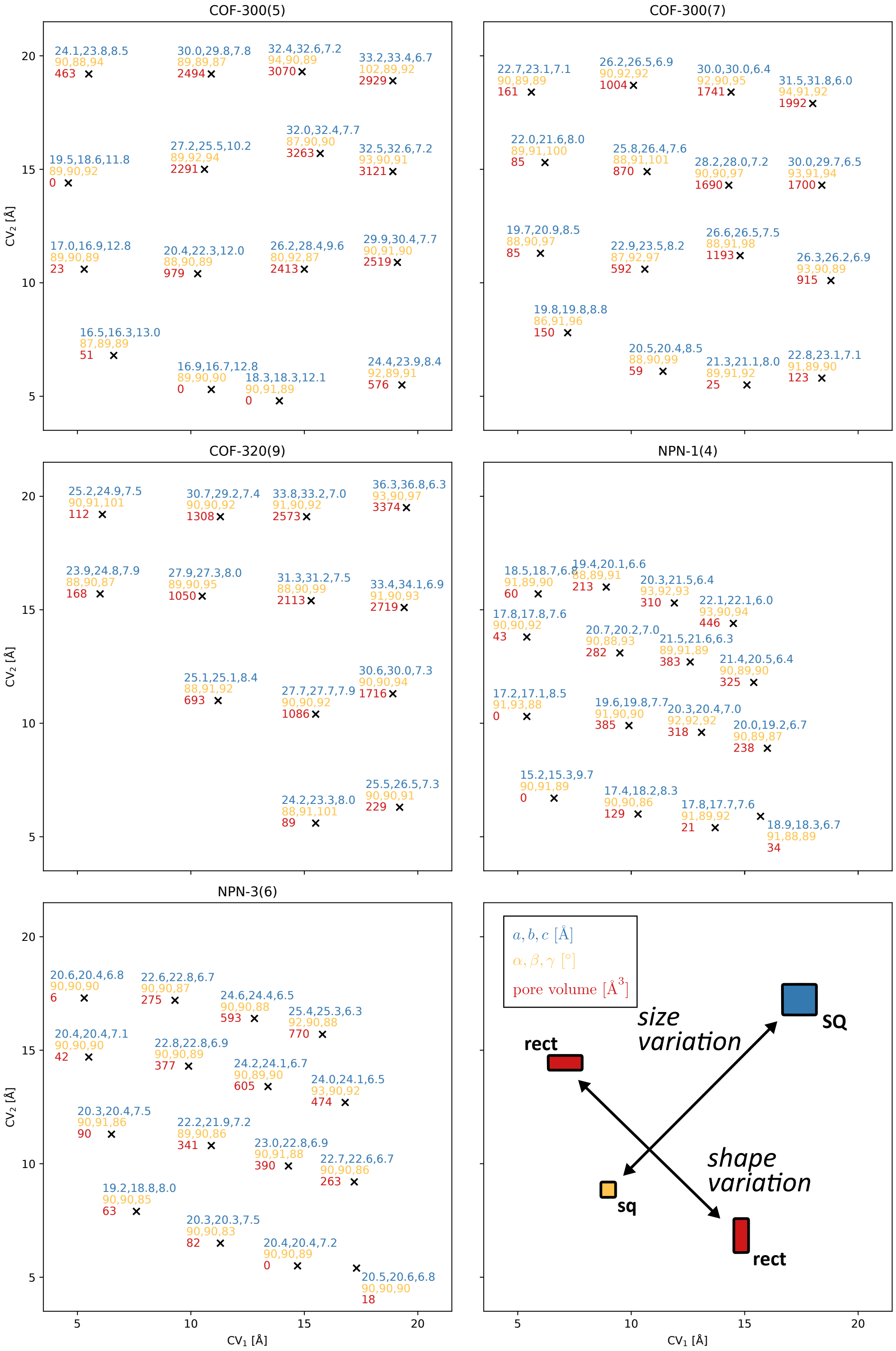

Supplementary Figure 2: Relation between the collective variables and the cell parameters for the materials of Table 1. The annotation for each data point corresponds to the legend in the bottom right.

Supplementary Note 4 Iterative WHAM algorithm

As mentioned in the main text, the WHAM algorithm from Grossfield *et al.*[10] failed in situations where the total sampled phase space was not rectangular. This was mainly problematic for those systems with a high degree of interpenetration, as steric hindrance prevented sampling in specific areas of the phase space. This is illustrated in Supplementary Figure 3, which visualises the density map over all umbrella sampling simulations for elevenfold interpenetrated COF-320 at 300 K, with a typical 'I'-shape. By exploiting the specific shape of the density maps, and the symmetry between the two collective variables, an iterative algorithm was created that varies the lower and upper edges provided to the WHAM code, and removes the simulations with a trajectory outside of these bounds, until an optimal set of rectangular regions is found that maximally resembles the sampled phase space. Afterwards, all free energy regions are related to each other using a common point (if it exists) or a daisy chaining algorithm (where each region is related to at least one other region). Evidently, both approaches require that the total free energy region is connected, as for any WHAM calculation. Finally, the total free energy at any given point is calculated as an average over its value for all regions in which it is defined.

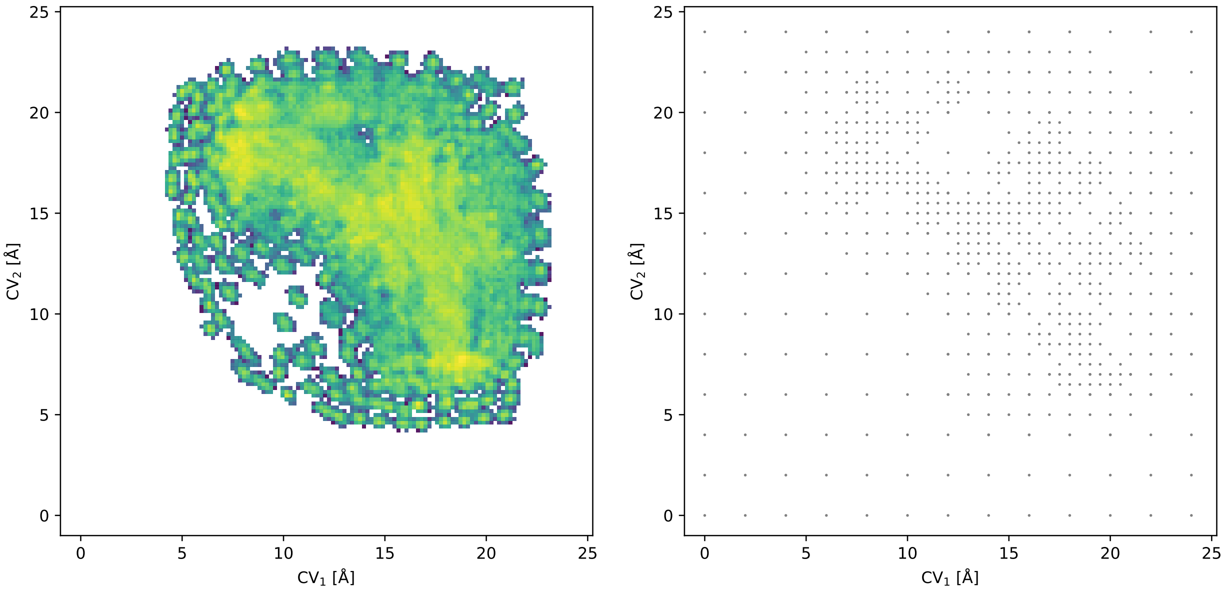

(a) Logarithmic density map. **(b)** Umbrella grid.

Supplementary Figure 3: Logarithmic density map over all umbrella sampling simulations for elevenfold interpenetration COF-320 at 300 K. A equilibration time of 5 ps was considered for all simulations, resulting in a typical 'I'-shaped density map.

A flowchart demonstrating the iterative algorithm is visualised in Supplementary Figure 5. In the first step, the optimal square edges are calculated, for which the lower and upper bound are identical for both CVs. When removing the trajectories with data outside the edges does not allow for a successful WHAM run, the lower bound will be consistently increased by 'step' until it reaches the provided maximum lower bound (maximum min_CV). At that point, the upper bound is decreased by 'step'. This process is repeated until a successful WHAM run is encountered or the upper bound becomes lower than the provided minimum upper bound (minimum max_CV). Then, all symmetrically inequivalent rectangular edges are iterated over, between the minimum and maximum CV values, skipping those edges that do not cover any additional surface and hence are redundant. Finally, all successful combinations (and their symmetrical equivalent) are combined in step 3 to create a full free energy surface.

In addition to this procedure, and as a validation, the in-house WHAM code from ThermoLIB was adapted to prevent the aforementioned convergence error, which originated in Boltzmann factors becoming infinitely small in regions with prohibitively low unbiased probabilities. As the WHAM algorithm requires a rectangular region, and the steric hindrance inhibits small channel sizes with increasing interpenetration, it is to be expected the WHAM algorithm fails without accounting for these problematic grid locations. By removing the responsible simulations, and masking the relevant grid locations, similar to the iterative procedure, only a single WHAM calculation is required to obtain the full profile. A comparison between the two processes for the density map in Supplementary Figure 3 is given in Supplementary Figure 4, showing an almost perfect agreement.

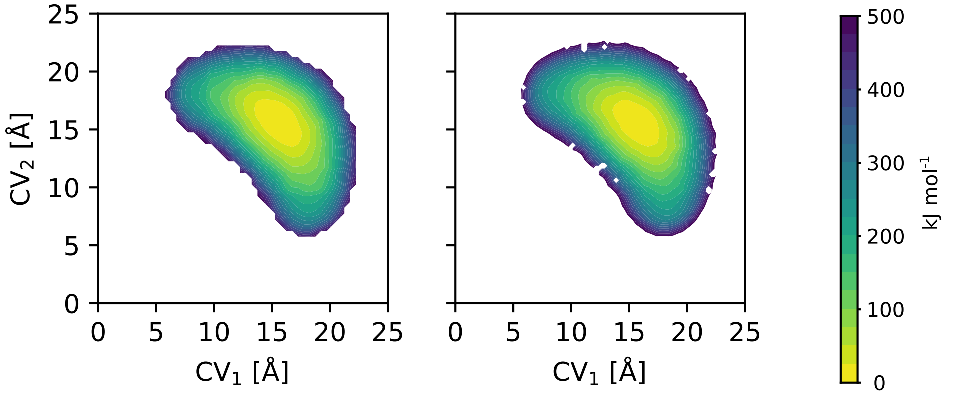

Supplementary Figure 4: Comparison between the free energy landscapes calculated with (left) the iterative procedure and (right) the ThermoLIB code for elevenfold interpenetrated COF-320 at 300 K.

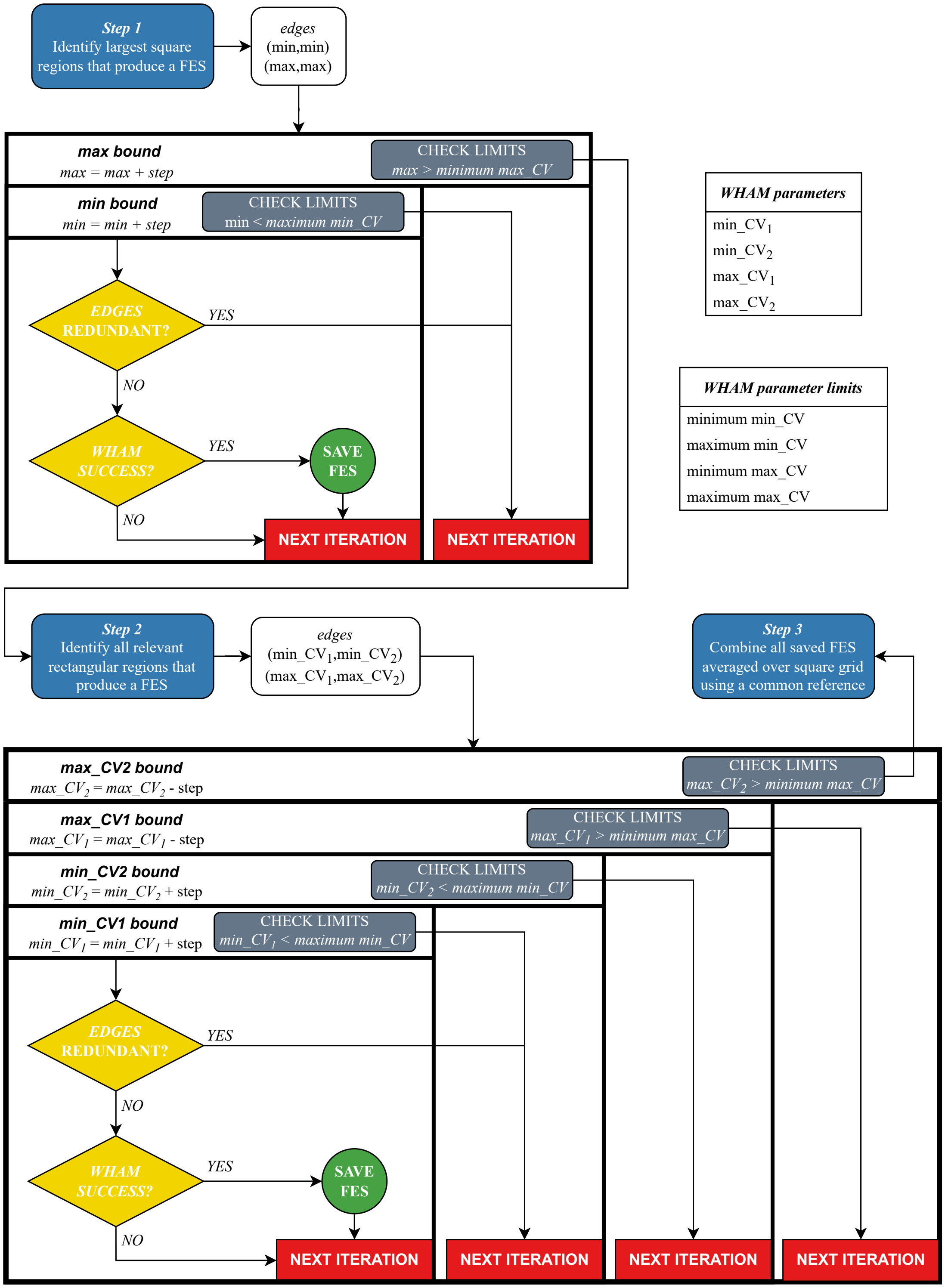

Supplementary Figure 5: Flowchart indicating the three steps that constitute the adaptive WHAM algorithm.

Supplementary Note 5 Free energy landscapes

Supplementary Note 5.1 Empty framework

The free energy landscapes as a function of interpenetration and temperature can be calculated for all materials, as captured in Supplementary Figures 6-15, for COF-300, COF-320, NPN-1, and NPN-3, using a temperature of either 50 K, 100 K, 300 K or 400 K. An overview of the number of simulations for each combination of material, degree of interpenetration, and temperature can be found in Tables 2-5. Note that the number of simulations generally decreases with an increasing degree of interpenetration due to the steric hindrance limiting the accessible phase space. Moreover, to limit the number of calculations, not all possible degrees of interpenetrations were considered for each material and temperature combination, aside for the reference calculations at 300 K. For the other temperatures, the non-interpenetrated framework was not considered due to a lack of stabilizing dispersive interactions, whereas the highest degrees of interpenetration were not considered due to the steric hindrance, both enforcing a single stable phase.

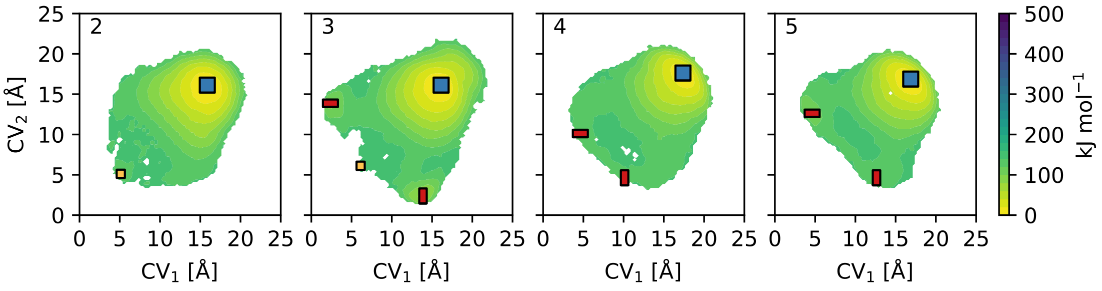

Supplementary Figure 6: Free energy surfaces for COF-300 at 50 K as a function of interpenetration, going from twofold to sevenfold interpenetrated, as indicated in the top left corner of each subpanel. The different (meta)stable phases are indicated on the figure. Regions with a free energy exceeding the free energy minimum by more than 500 kJ mol^{-1} are omitted from the plot, as they are not accessible under realistic conditions.

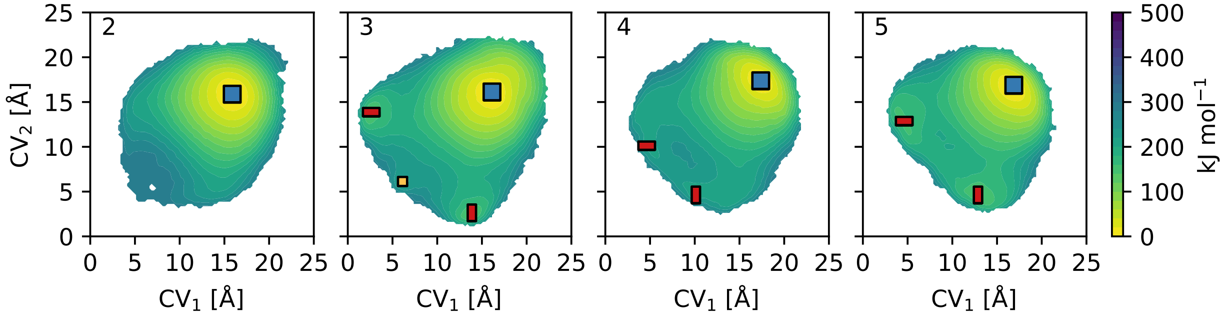

Supplementary Figure 7: Free energy surfaces for COF-300 at 100 K as a function of interpenetration, going from twofold to sevenfold interpenetrated, as indicated in the top left corner of each subpanel. The different (meta)stable phases are indicated on the figure. Regions with a free energy exceeding the free energy minimum by more than 500 kJ mol^{-1} are omitted from the plot, as they are not accessible under realistic conditions.

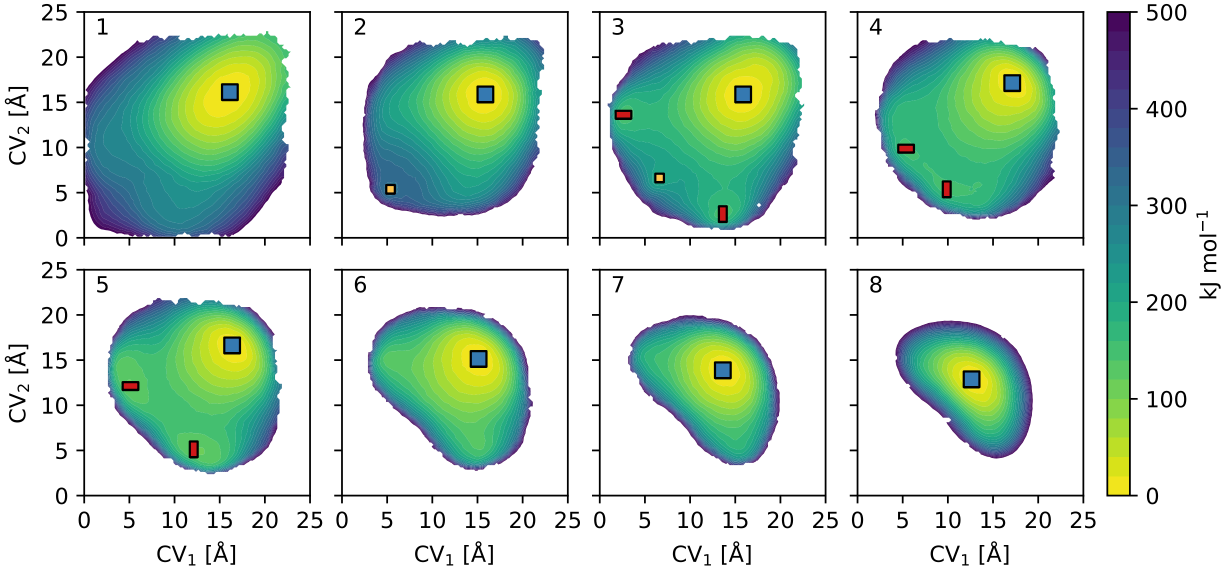

Supplementary Figure 8: Free energy surfaces for COF-300 at 300 K as a function of interpenetration, going from non-interpenetrated to eightfold interpenetrated, as indicated in the top left corner of each subpanel. The different (meta)stable phases are indicated on the figure. Regions with a free energy exceeding the free energy minimum by more than 500 kJ mol^{-1} are omitted from the plot, as they are not accessible under realistic conditions.

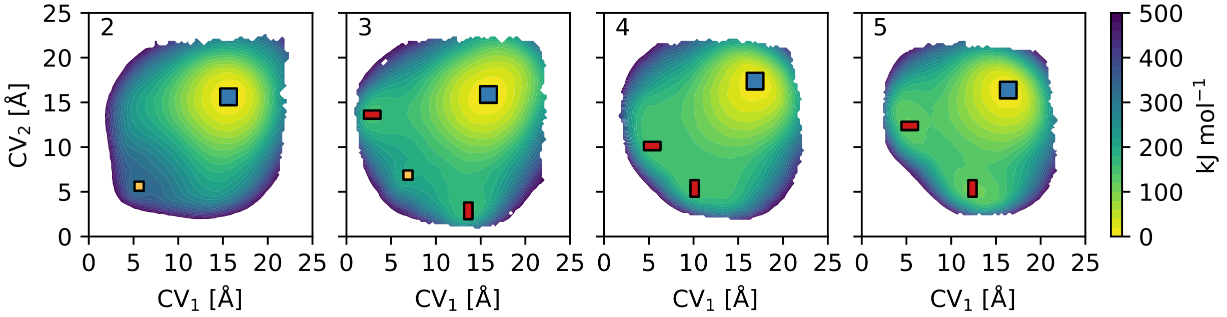

Supplementary Figure 9: Free energy surfaces for COF-300 at 400 K as a function of interpenetration, going from twofold to sixfold interpenetrated, as indicated in the top left corner of each subpanel. The different (meta)stable phases are indicated on the figure. Regions with a free energy exceeding the free energy minimum by more than 500 kJ mol^{-1} are omitted from the plot, as they are not accessible under realistic conditions.

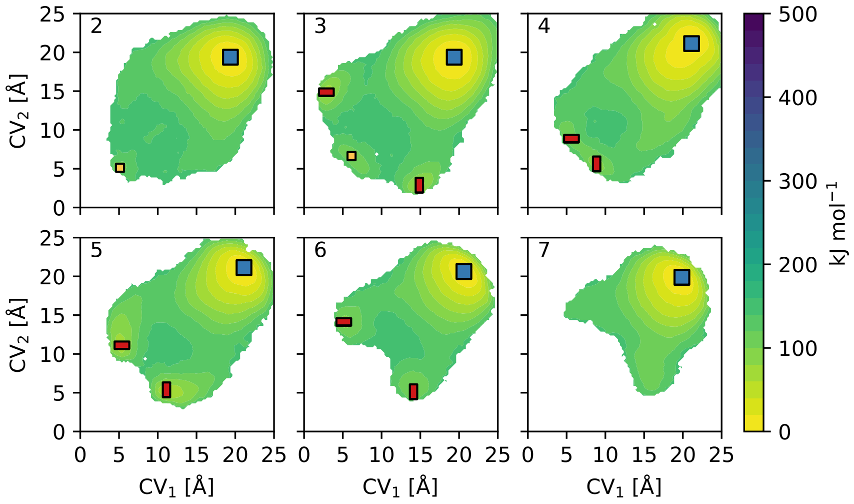

Supplementary Figure 10: Free energy surfaces for COF-320 at 50 K as a function of interpenetration, showcasing twofold, fivefold, and ninefold interpenetrated, as indicated in the top left corner of each subpanel. The different (meta)stable phases are indicated on the figure. Regions with a free energy exceeding the free energy minimum by more than 500 kJ mol^{-1} are omitted from the plot, as they are not accessible under realistic conditions.

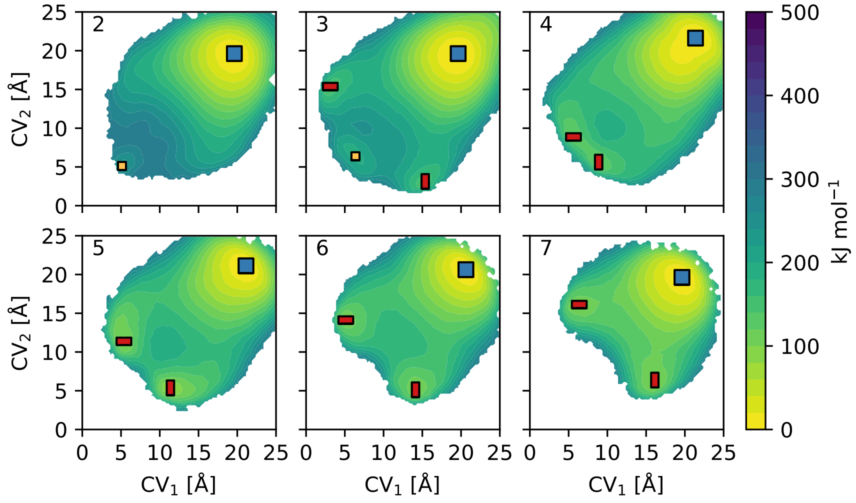

Supplementary Figure 11: Free energy surfaces for COF-320 at 100 K as a function of interpenetration, showcasing twofold, fivefold, and ninefold interpenetrated, as indicated in the top left corner of each subpanel. The different (meta)stable phases are indicated on the figure. Regions with a free energy exceeding the free energy minimum by more than 500 kJ mol^{-1} are omitted from the plot, as they are not accessible under realistic conditions.

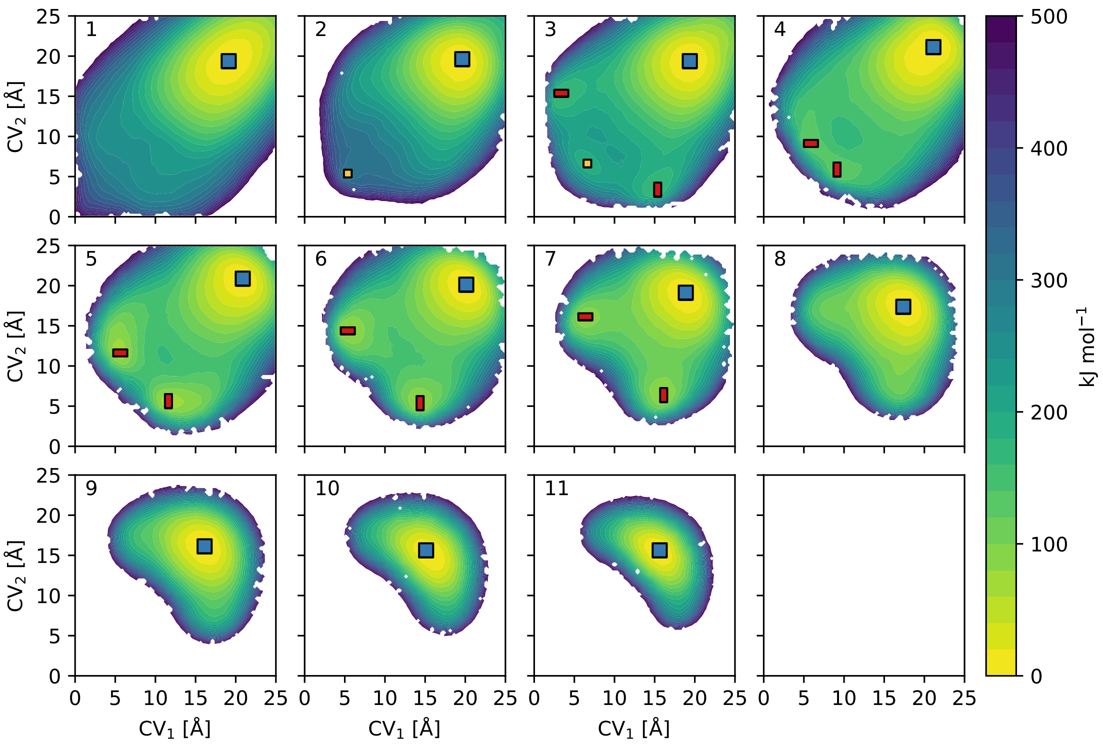

Supplementary Figure 12: Free energy surfaces for COF-320 at 300 K as a function of interpenetration, going from non-interpenetrated to elevenfold interpenetrated, as indicated in the top left corner of each subpanel. The different (meta)stable phases are indicated on the figure. Regions with a free energy exceeding the free energy minimum by more than 500 kJ mol^{-1} are omitted from the plot, as they are not accessible under realistic conditions.

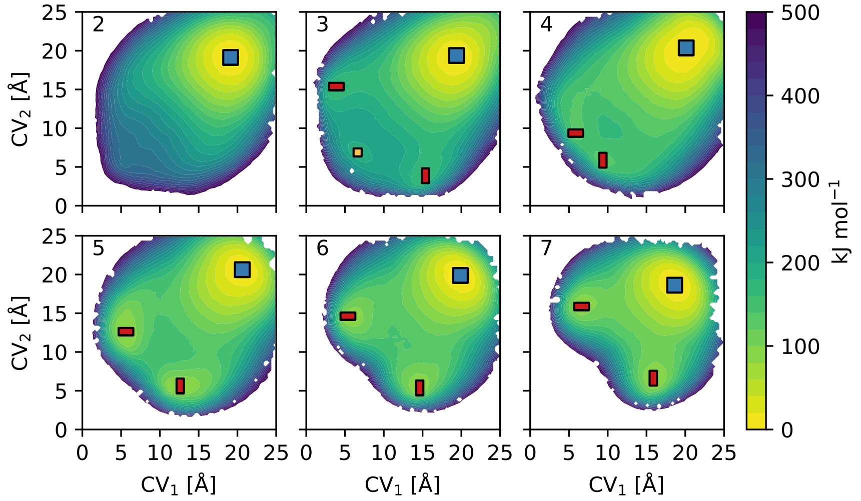

Supplementary Figure 13: Free energy surfaces for COF-320 at 400 K as a function of interpenetration, showcasing twofold, fivefold, and ninefold interpenetrated, as indicated in the top left corner of each subpanel. The different (meta)stable phases are indicated on the figure. Regions with a free energy exceeding the free energy minimum by more than 500 kJ mol^{-1} are omitted from the plot, as they are not accessible under realistic conditions.

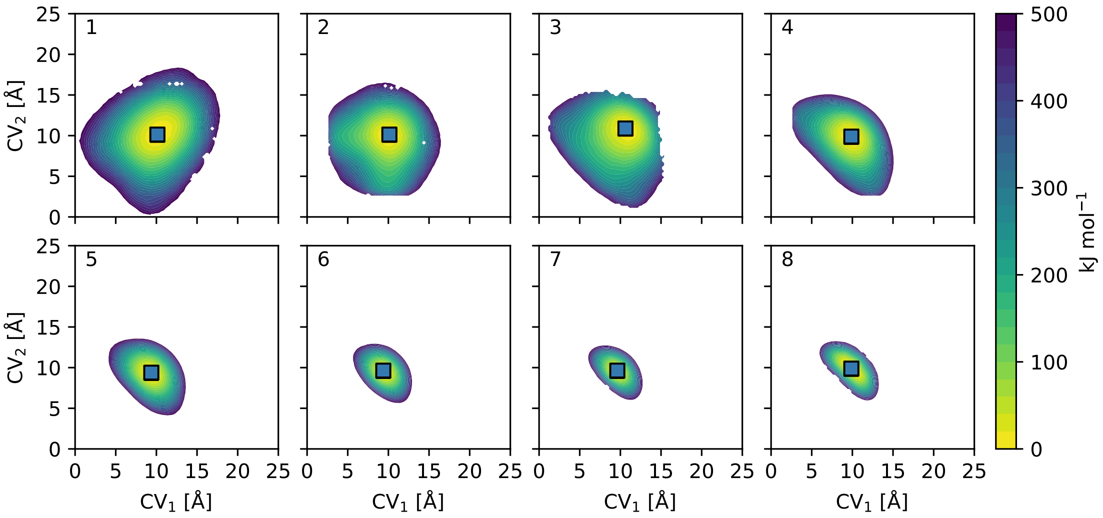

Supplementary Figure 14: Free energy surfaces for NPN-1 at 300 K as a function of interpenetration, going from non-interpenetrated to eightfold interpenetrated, as indicated in the top left corner of each subpanel. The different (meta)stable phases are indicated on the figure. Regions with a free energy exceeding the free energy minimum by more than 500 kJ mol^{-1} are omitted from the plot, as they are not accessible under realistic conditions.

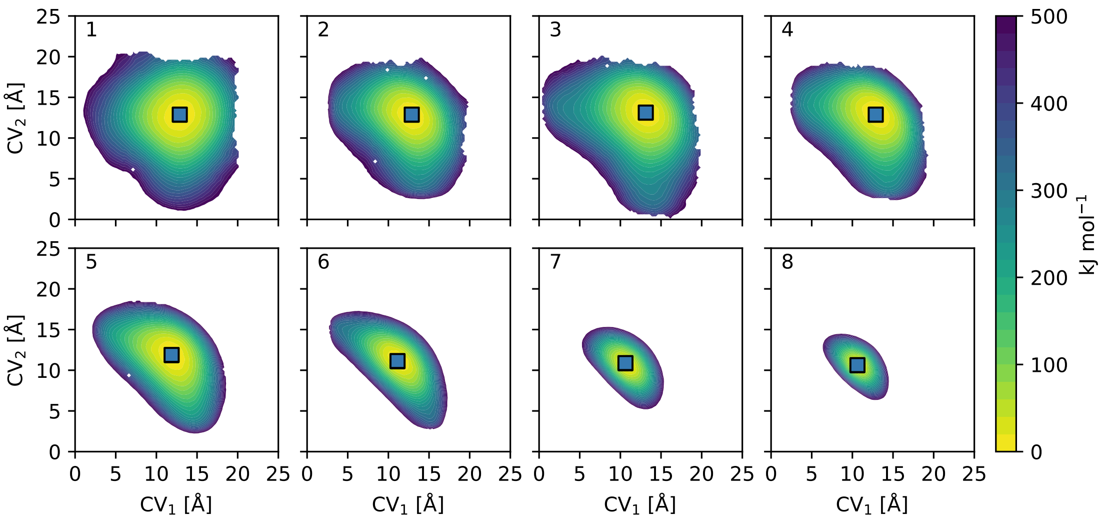

Supplementary Figure 15: Free energy surfaces for NPN-3 at 300 K as a function of interpenetration, going from non-interpenetrated to eightfold interpenetrated, as indicated in the top left corner of each subpanel. The different (meta)stable phases are indicated on the figure. Regions with a free energy exceeding the free energy minimum by more than 500 kJ mol^{-1} are omitted from the plot, as they are not accessible under realistic conditions.

Supplementary Table 2: Number of umbrella sampling simulations at unique grid points as a function of the degree of interpenetration for the considered materials at 50 K.

	degree of interpenetration										
	1fold	2fold	3fold	4fold	5fold	6fold	7fold	8fold	9fold	10fold	11fold
COF-300		1807	1937	1778	1657						
COF-320		2542	2632	2430	2299	2097	1918				

Supplementary Table 3: Number of umbrella sampling simulations at unique grid points as a function of the degree of interpenetration for the considered materials at 100 K.

	degree of interpenetration										
	1fold	2fold	3fold	4fold	5fold	6fold	7fold	8fold	9fold	10fold	11fold
COF-300		1843	1940	1727	1612						
COF-320		2422	2525	2372	2235	2059	1894				

Supplementary Table 4: Number of umbrella sampling simulations at unique grid points as a function of the degree of interpenetration for the considered materials at 300 K.

	degree of interpenetration										
	1fold	2fold	3fold	4fold	5fold	6fold	7fold	8fold	9fold	10fold	11fold
COF-300	2144	1750	1843	1644	1521	1306	1183	905			
COF-320	2716	2462	2341	2298	2172	1907	1717	1521	1250	939	866
NPN-1	957	859	760	688	531	510	519	531			
NPN-3	1583	1152	1361	1088	903	744	574	586			

Supplementary Table 5: Number of umbrella sampling simulations at unique grid points as a function of the degree of interpenetration for the considered materials at 400 K.

	degree of interpenetration										
	1fold	2fold	3fold	4fold	5fold	6fold	7fold	8fold	9fold	10fold	11fold
COF-300		1784	1884	1648	1496						
COF-320		2416	2363	2303	2146	1922	1732				

Supplementary Note 5.2 Water-filled framework

Similar to the empty frameworks, the free energy landscapes can also be considered as a function of the water loading, for a fixed degree of interpenetration and temperature. To validate the reported COF-300(7) behaviour when adsorbing water, the loading is exponentially varied from one to sixteen water molecules per channel, revealing a volume contraction upon water adsorption. While the observed unit cell volumes values differ somewhat from those observed experimentally, as tabulated in Table 6, the relative change in volume upon water adsorption (-6%) is very similar to the values from those references mentioned in Table 1, succeeding in a qualitative description of the experimentally observed pore shrinkage upon water adsorption.

Supplementary Table 6: Water induced volume changes for COF-300(7) compared between this work and experimental reference data. A loading of eight water molecules is assumed for the hydrated volume in this work.

	$V_{hydrated}$ [Å^3]	V_{empty} [Å^3]	$\Delta V/V$ [%]
This work	5275	5594	-6
[4]	3435	5210	-34
[5]	3433	3644	-6
[6]	3683	3824	-4

Supplementary Note 5.3 Derivation of transition barriers

Through application of the Minimum Energy Path Surface Analysis (MEPSA) code,[11] which requires the FES as input data, the individual local minima can be extracted from the energy landscape, as illustrated in the left panel of Supplementary Figure 16. By analysing the resulting minimal free energy paths between the identified minima (right panel), we can calculate the transition barrier heights by calculating the difference between the free energy of the (meta)stable phase and the neighbouring local maximum, that delineates the basin of attraction for that specific phase.

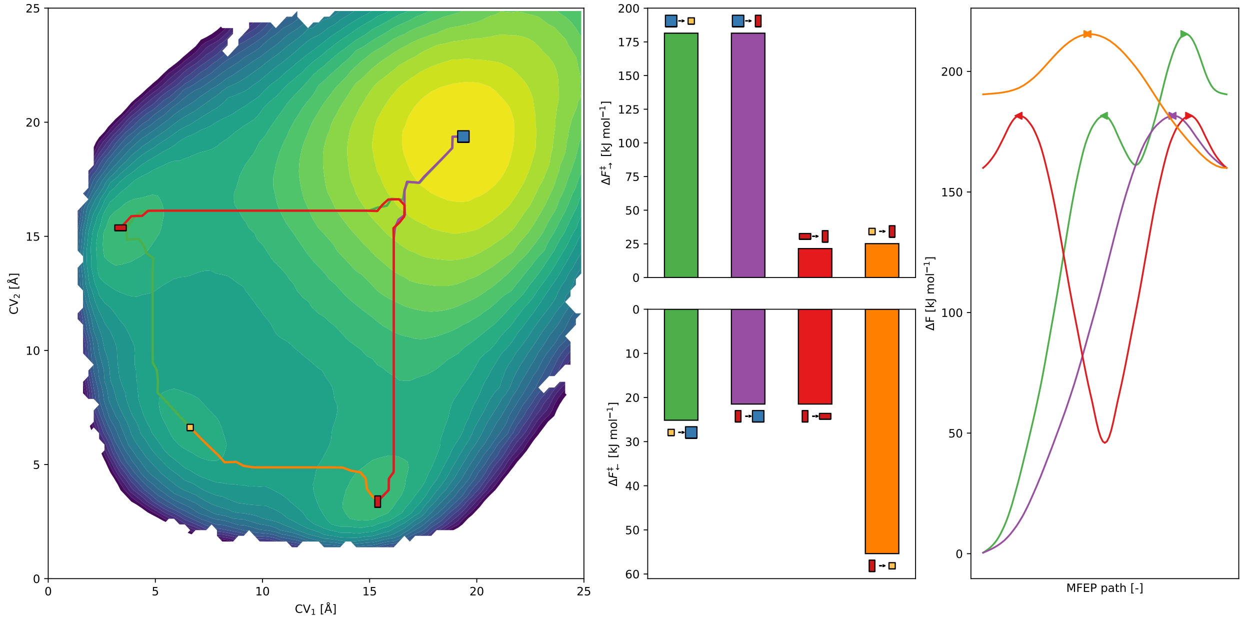

Supplementary Figure 16: Analysis of the transition height barriers for threefold interpenetrated COF-320 at 300 K using MEPSA. (Left) Identification of local minima as the (meta)stable phases. (Middle) Forwards and backwards transition barriers as derived from the free energy paths drawn in the left panel. (Right) The 1D free energy paths corresponding to the paths drawn in the left panel.

Supplementary Note 6 From *ab initio* cluster structures to periodic force field structures

Supplementary Note 6.1 Generation of cluster models

Following our general accepted approach to feasibly estimate force field parameters for periodic materials,[12–20] the framework structure is decomposed into clusters that each mimic a part of the framework and their environment. Although this clustering procedure results in a considerably reduced calculation time for the *ab initio* calculations required to derive a force field, the termination of each cluster should be carefully considered, as the optimal cluster geometry might deviate significantly from its periodic equivalent. When cutting and saturating the bonds at the edges of the cluster model in a suboptimal way, artificial charges and/or spins can be induced, altering the desired molecular environment. Here the cluster models were terminated with phenyl rings to optimally mimic the molecular environment of the cluster, as visualised in Supplementary Figure 17. COF-300 and COF-320 were decomposed into two clusters (a/c and b/c), whereas NPN-1 and NPN-3 were approximated by a single cluster (d and e), similar to their synthesis procedure.

The cluster geometries were optimised with the Gaussian16 suite,[21] using the B3LYP exchange-correlation functional[22–24] extended with the Grimme-D3 dispersion corrections[25] and the 6-311++G(d,p) Pople basis set.[26] For convergence, the default criteria for distances and angles were adopted (maximum force=0.00045 Ha/a_0 and Ha/rad, rms force=0.00030 Ha/a_0 and Ha/rad, maximum displacement=0.00180 a_0 and rad, rms displacement=0.00120 a_0 and rad). Subsequently, the Hessian was calculated and a normal mode analysis was performed to validate whether no negative frequencies were present. Then, from the equilibrium electron density, the atomic partial charges were calculated with the MBIS partitioning scheme,[27] as implemented in HORTON.[28]

These *ab initio* data were then provided as input to the QuickFF protocol,[29,30] first, to derive the electrostatic force field parameters from the MBIS charges, using a Gaussian charge distribution[31] and bond charge increments,[32] and second, to derive the covalent force field parameters. For the latter, van der Waals force field parameters were included during the fitting procedure, using the MM3-Buckingham potential as parametrized by Allinger *et al.*,[33] since the *ab initio* calculations were supplemented with the Grimme-D3 dispersion corrections. The cluster force fields have been provided as additional supplementary files, and can be found at `https://github.com/SanderBorgmans/SupportingInformation`.

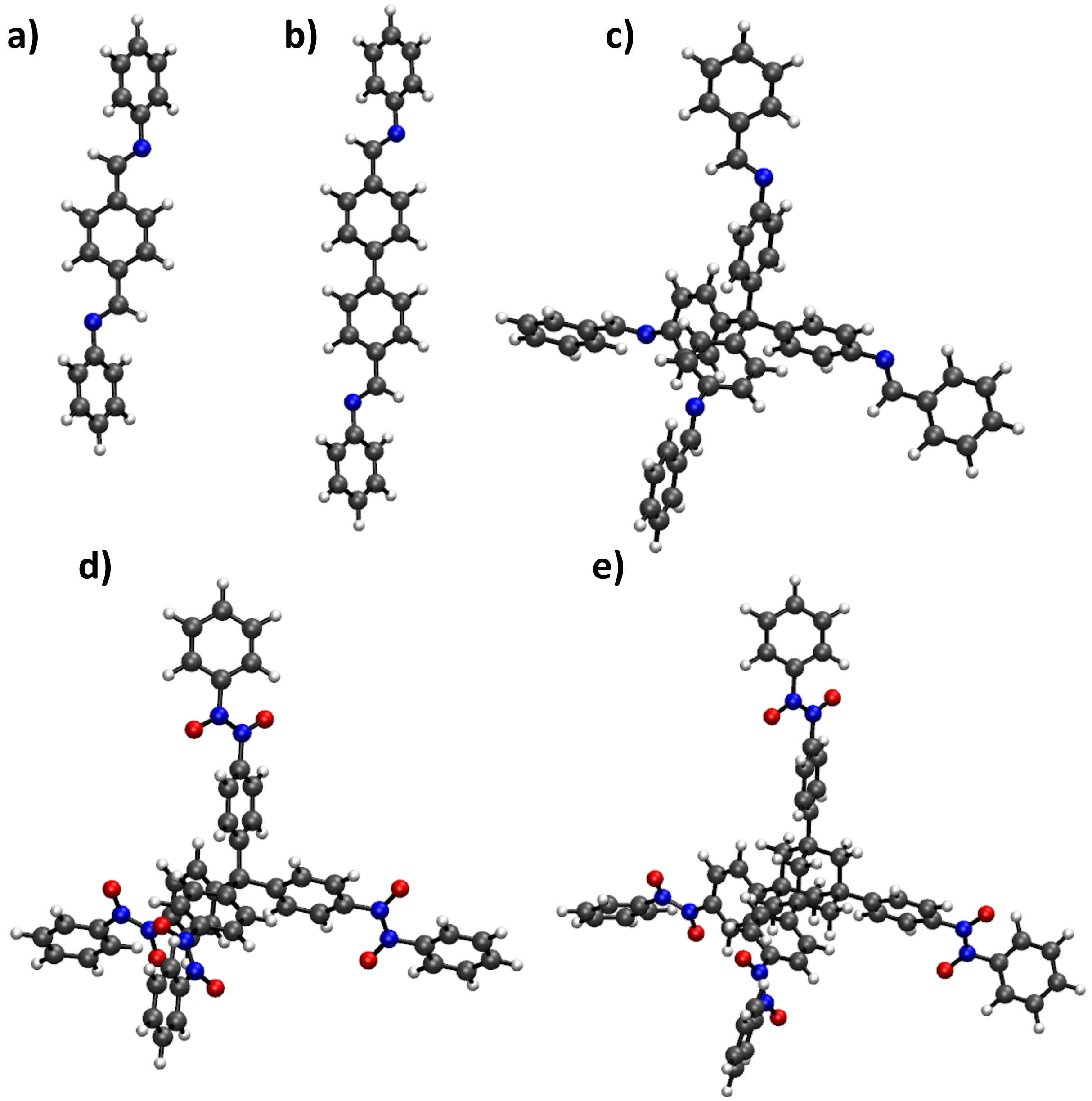

Supplementary Figure 17: Cluster models used in the *ab initio* calculations for (a) terephthalaldehyde, (b) 4-(4-formylphenyl)benzaldehyde, (c) tetra-(4-anilyl)methane, (d) tetrakis(4-nitrosophenyl)methane, and (e) 1,3,5,7-tetrakis(4-nitrosophenyl)adamantane. All clusters were terminated with phenyl rings to optimally mimic the molecular environment of the clusters in the periodic material. The following colour code was used: (white) hydrogen, (grey) carbon, (blue) nitrogen, (red) oxygen.

Supplementary Note 6.2 Validation procedure

The cluster force fields were validated through a vibrational frequency analysis, performed with TAMkin.[34] This constitutes the comparison of the *ab initio* and force field frequencies, as calculated with the normal mode analysis (NMA) technique.[35] This provides a quantitative measure for the accuracy of the fitting procedure of the force field. The mean deviation (MD) and root mean square deviations (RMSD) have been indicated in Supplementary Figure 18 for each cluster model, demonstrating a good correspondence.

Aside from the frequency comparison, the optimal internal coordinates for the force fields are also compared with respect to the *ab initio* reference values, as visualised in Supplementary Figure 19. Again, the error made by the force fields is quantified through the MD and RMSD.

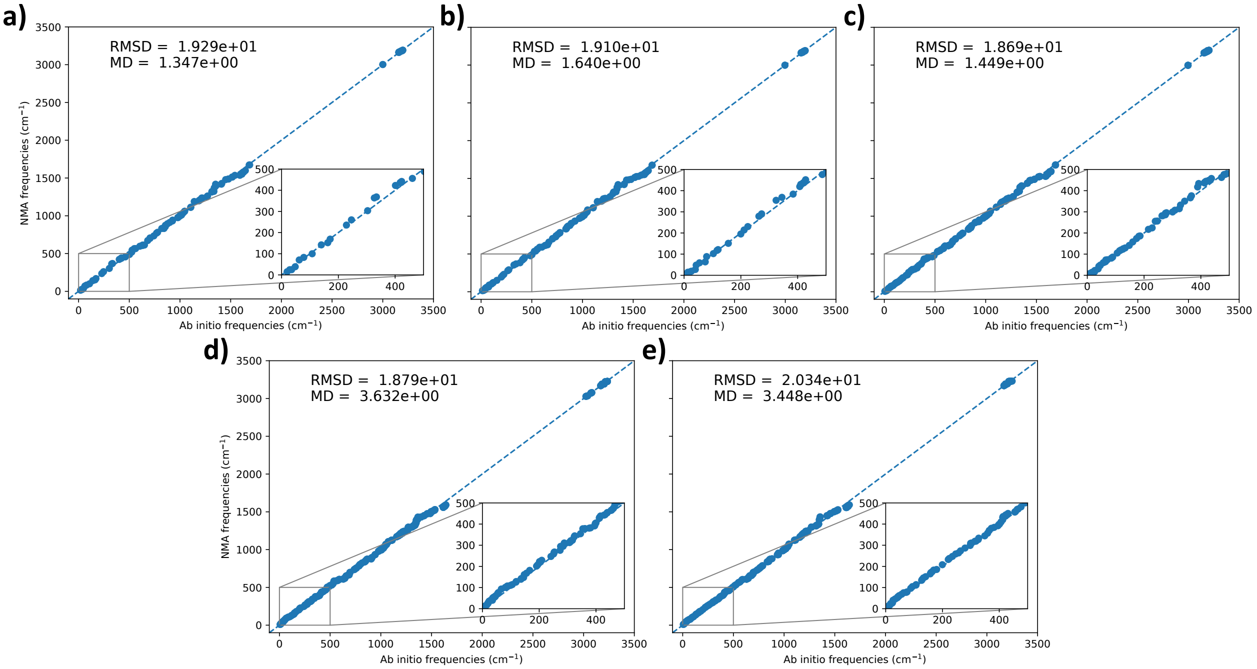

Supplementary Figure 18: Comparison of the *ab initio* (AI) and force field (FF) NMA frequencies for all the cluster models, with a zoomed picture for the low frequencies in the bottom right corner. The dashed lines indicate a perfect agreement. The alphabetical labels correspond to Supplementary Figure 17.

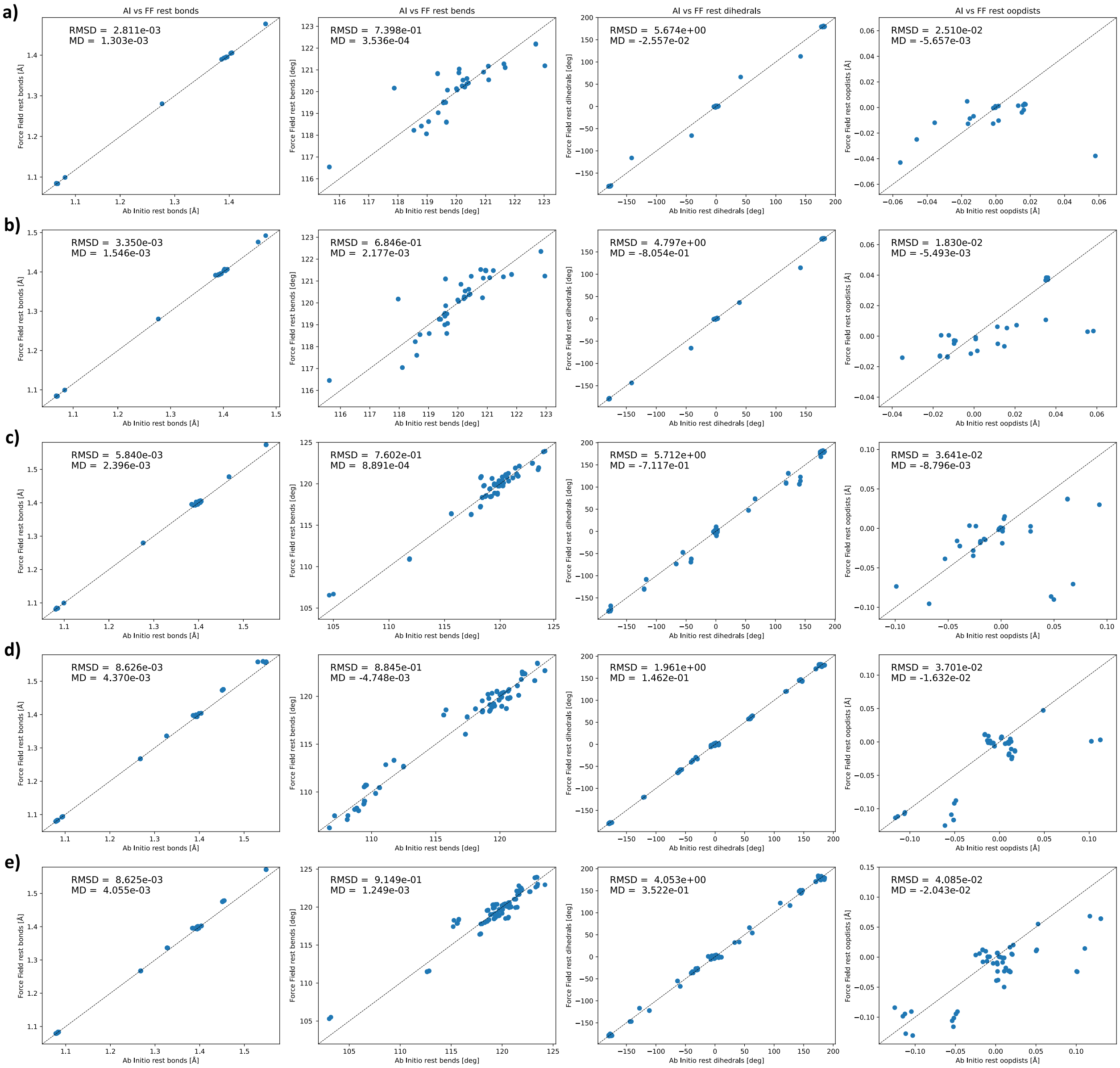

Supplementary Figure 19: Comparison of the *ab initio* (AI) and force field (FF) internal coordinates. The dashed lines indicate a perfect agreement. The alphabetical labels correspond to Figure 17.

Supplementary Note 6.3 Combining cluster force fields

Since COF-300 and COF-320 both consist of two clusters, the respective cluster force fields have to be combined into a single force field for the periodic material. To this end, the atom types of each cluster are divided into a 'core' and 'termination' category, in correspondence with the aforementioned decomposition of the periodic material into representative parts and their surroundings. The force field parameters for the periodic force field are subsequently calculated as a weighted average from the cluster force field parameters, where the weight for each term is equal to the amount of 'core' atom types. This workflow assumes that the parameters contained in the 'core' region are fitted to their optimal value in the periodic material, whereas the termination 'region' might deviate significantly. All resulting combined force fields are provided as supplementary files, found on the previously mentioned GitHub page.

Supplementary Note 6.4 Additional force field terms

The main deviations in the internal coordinates (Supplementary Figure 19) stem from the imine linkages in the cluster models for COF-300 and COF-320. Moreover, the corresponding rotational barrier predicted by the force field deviates significantly from the *ab initio* reference, as observed in the left panels of Supplementary Figures 20-21, for COF-300 and COF-320. This can be accommodated by introducing additional force field terms, replacing the responsible dihedral term. Here, a sixth order polynomial as a function of the cosine of the dihedral angle is used, removing all odd powers to account for the rotational symmetry, and discarding the irrelevant constant term:

$$V_{dih}(\cos(\phi)) = c_0 \cos^2(\phi) + c_1 \cos^4(\phi) + c_2 \cos^6(\phi) \tag{S6.1}$$

For COF-300, the fitting procedure results in $c_0 = -16.9$ kJ mol^{-1}, $c_1 = 11.5$ kJ mol^{-1}, and $c_2 = -7.5$ kJ mol^{-1}, whereas for COF-320, this results in $c_0 = -16.9$ kJ mol^{-1}, $c_1 = 12.9$ kJ mol^{-1}, and $c_2 = -8.5$ kJ mol^{-1}. The similarity between the two sets of parameters can be expected, since the molecular environment of the imine linkage is quasi identical. As evident from Supplementary Figures 20-21, the additional terms result in a near perfect reproduction of the *ab initio* behaviour, as the red and purple graphs coincide.

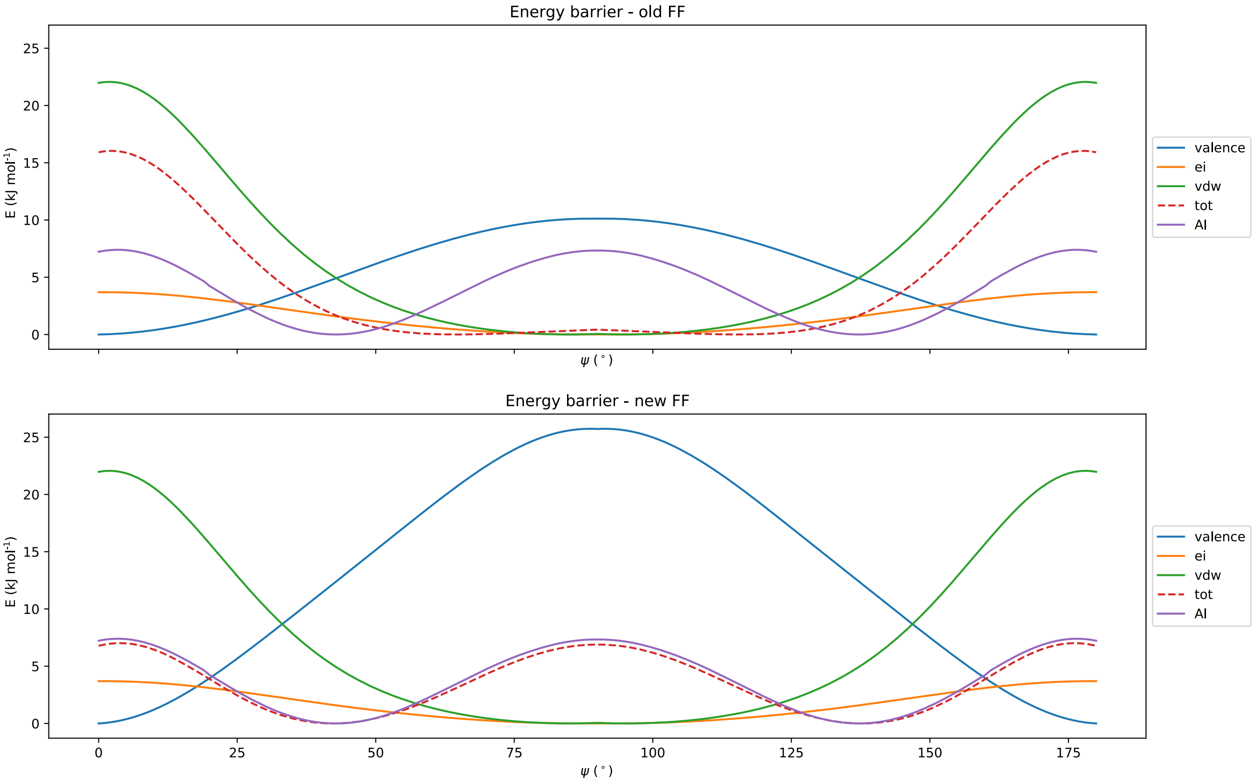

Supplementary Figure 20: Force field contributions for the rotation of the imine linked building block of COF-300, described by the dihedral angle ϕ. The total force field energy (tot) is equal to the sum of the covalent contributions (valence), the electrostatic interactions (ei) and the dispersion interactions (vdw), and should reproduce the *ab initio* energy scan (AI). (Left) Original force field. (Right) Adapted force field, with additional sixth order polynomial for the covalent contributions expressed in Equation S6.1.

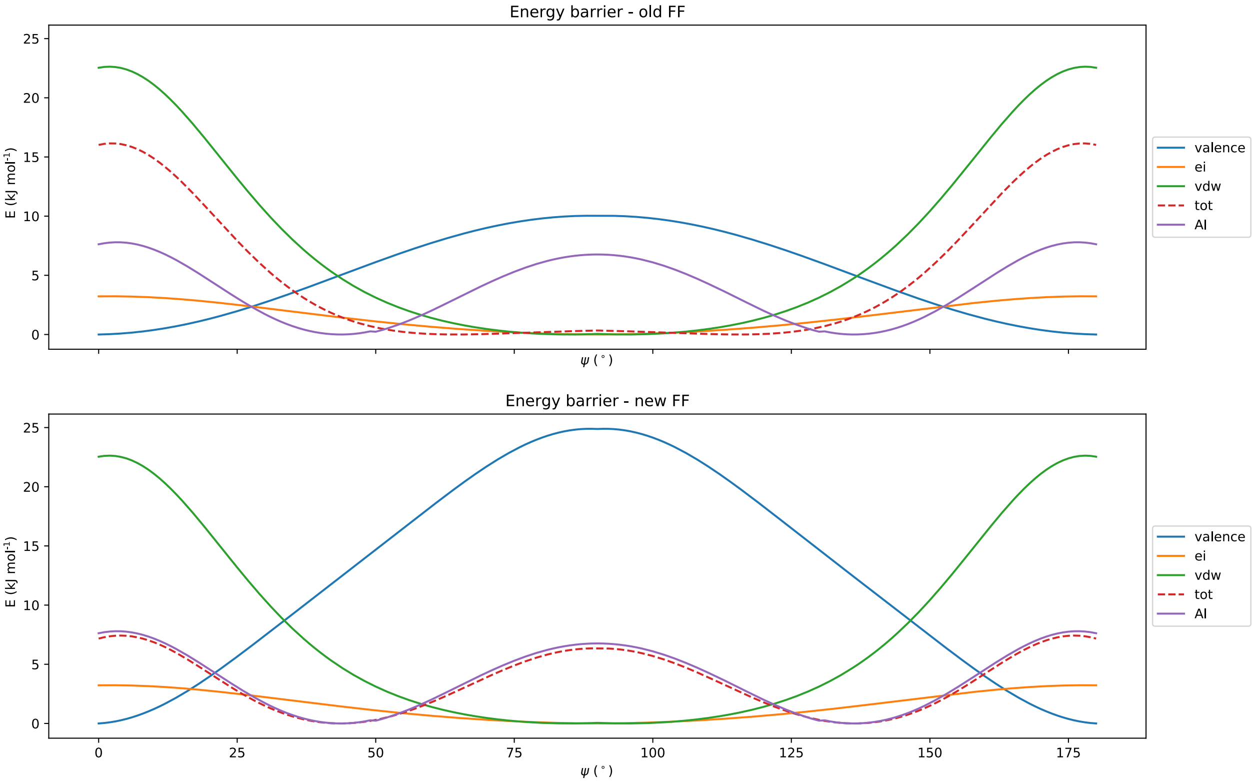

Supplementary Figure 21: Force field contributions for the rotation of the imine linked building block of COF-320, described by the dihedral angle ϕ. The total force field energy (tot) is equal to the sum of the covalent contributions (valence), the electrostatic interactions (ei) and the dispersion interactions (vdw), and should reproduce the *ab initio* energy scan (AI). (Left) Original force field. (Right) Adapted force field, with additional sixth order polynomial for the covalent contributions expressed in Equation S6.1.

Supplementary Note 6.5 Modelling water-framework interactions

As the original periodic framework force fields did not yet account for the water-water and water-framework interactions, these interaction parameters were added *a posteriori*. The water-water interactions were reproduced from ref. 6, based on the TIP4P/2005f force field from ref. 36, using a Lennard-Jones potential and point-charge electrostatics. Then, to capture the interactions between the water molecules and the framework atoms, individual Lennard-Jones cross terms were derived, as the framework-framework interactions were defined in terms of the MM3-Buckingham parameters of Allinger *et al.*[33] To this end, the Lennard-Jones parameters for the framework atoms were reproduced from the UFF force field,[37] and the relevant cross terms were calculated using the default mixing rules:

$$\sigma_{ij} = \frac{\sigma_i + \sigma_j}{2} \qquad \text{and} \qquad \epsilon_{ij} = \sqrt{\epsilon_i \epsilon_j} \tag{S6.2}$$

As such, introducing these mixing rules ensures that the framework-framework and water-water interactions are unchanged with respect to the original framework calculations and the original water simulations.

Supplementary References

(1) Zhang, Y.-B.; Su, J.; Furukawa, H.; Yun, Y.; Gándara, F.; Duong, A.; Zou, X.; Yaghi, O. M. *J. Am. Chem. Soc.* **2013**, *135*, 16336–16339.

(2) Beaudoin, D.; Maris, T.; Wuest, J. D. *Nat. Chem.* **2013**, *5*, 830–834.

(3) Uribe-Romo, F. J.; Hunt, J. R.; Furukawa, H.; Klöck, C.; O'Keeffe, M.; Yaghi, O. M. *J. Am. Chem. Soc.* **2009**, *131*, 4570–4571.

(4) Ma, T.; Kapustin, E. A.; Yin, S. X.; Liang, L.; Zhou, Z.; Niu, J.; Li, L.-H.; Wang, Y.; Su, J.; Li, J.; Wang, X.; Wang, W. D.; Wang, W.; Sun, J.; Yaghi, O. M. *Science* **2018**, *361*, 48–52.

(5) Chen, Y.; Shi, Z.-L.; Wei, L.; Zhou, B.; Tan, J.; Zhou, H.-L.; Zhang, Y.-B. *J. Am. Chem. Soc.* **2019**, *141*, 3298–3303.

(6) Sun, T.; Wei, L.; Chen, Y.; Ma, Y.; Zhang, Y.-B. *J. Am. Chem. Soc.* **2019**, *141*, 10962–10966.

(7) Ma, T.; Li, J.; Niu, J.; Zhang, L.; Etman, A. S.; Lin, C.; Shi, D.; Chen, P.; Li, L.-H.; Du, X.; Sun, J.; Wang, W. *J. Am. Chem. Soc.* **2018**, *140*, 6763–6766.

(8) Martin, R. L.; Smit, B.; Haranczyk, M. *J. Chem. Inf. Model.* **2011**, *52*, 308–318.

(9) Willems, T. F.; Rycroft, C. H.; Kazi, M.; Meza, J. C.; Haranczyk, M. *Microporous Mesoporous Mater.* **2012**, *149*, 134–141.

(10) Grossfield, A. WHAM: The weighted histogram analysis method. `hhttp://membrane.urmc.rochester.edu/content/wham`.

(11) Marcos-Alcalde, I.; Setoain, J.; Mendieta-Moreno, J. I.; Mendieta, J.; Gómez-Puertas, P. *Bioinformatics* **2015**, *31*, 3853–3855.

(12) Tafipolsky, M.; Amirjalayer, S.; Schmid, R. *J. Comput. Chem.* **2007**, *28*, 1169–1176.

(13) Schmid, R.; Tafipolsky, M. *J. Am. Chem. Soc.* **2008**, *130*, 12600–12601.

(14) Tafipolsky, M.; Schmid, R. *J. Phys. Chem. B* **2009**, *113*, 1341–1352.

(15) Tafipolsky, M.; Amirjalayer, S.; Schmid, R. *J. Phys. Chem. C* **2010**, *114*, 14402–14409.

(16) Vanduyfhuys, L.; Verstraelen, T.; Vandichel, M.; Waroquier, M.; Van Speybroeck, V. *J. Chem. Theory Comput.* **2012**, *8*, 3217–3231.

(17) Amirjalayer, S.; Snurr, R. Q.; Schmid, R. *J. Phys. Chem. C* **2012**, *116*, 4921–4929.

(18) Bureekaew, S.; Amirjalayer, S.; Tafipolsky, M.; Spickermann, C.; Roy, T. K.; Schmid, R. *Phys. Status Solidi B* **2013**, *250*, 1128–1141.

(19) Rogge, S. M. J.; Wieme, J.; Vanduyfhuys, L.; Vandenbrande, S.; Maurin, G.; Verstraelen, T.; Waroquier, M.; Van Speybroeck, V. *Chem. Mater.* **2016**, *28*, 5721–5732.

(20) Wieme, J.; Vanduyfhuys, L.; Rogge, S. M. J.; Waroquier, M.; Van Speybroeck, V. *J. Phys. Chem. C* **2016**, *120*, 14934–14947.

(21) Frisch, M. J.; Trucks, G. W.; Schlegel, H. B.; Scuseria, G. E.; Robb, M. A.; Cheeseman, J. R.; Scalmani, G.; Barone, V.; Petersson, G. A.; Nakatsuji, H.; Li, X.; Caricato, M.; Marenich, A. V.; Bloino, J.; Janesko, B. G.; Gomperts, R.; Mennucci, B.; Hratchian, H. P.; Ortiz, J. V.; Izmaylov, A. F.; Sonnenberg, J. L.; Williams-Young, D.; Ding, F.; Lipparini, F.; Egidi, F.; Goings, J.; Peng, B.; Petrone, A.; Henderson, T.; Ranasinghe, D.; Zakrzewski, V. G.; Gao, J.; Rega, N.; Zheng, G.; Liang, W.; Hada, M.; Ehara, M.; Toyota, K.; Fukuda, R.; Hasegawa, J.; Ishida, M.; Nakajima, T.; Honda, Y.; Kitao, O.; Nakai, H.; Vreven, T.; Throssell, K.; Montgomery, J. A., Jr.; Peralta, J. E.; Ogliaro, F.; Bearpark, M. J.; Heyd, J. J.; Brothers, E. N.; Kudin, K. N.; Staroverov, V. N.; Keith, T. A.; Kobayashi, R.; Normand, J.; Raghavachari, K.; Rendell, A. P.; Burant, J. C.; Iyengar, S. S.; Tomasi, J.; Cossi, M.; Millam, J. M.; Klene, M.; Adamo, C.; Cammi, R.; Ochterski, J. W.; Martin, R. L.; Morokuma, K.; Farkas, O.; Foresman, J. B.; Fox, D. J. Gaussian 16 Revision A.03. 2016; Gaussian Inc. Wallingford CT.

(22) Becke, A. D. *J. Chem. Phys.* **1993**, *98*, 5648–5652.

(23) Lee, C.; Yang, W.; Parr, R. G. *Phys. Rev. B* **1988**, *37*, 785–789.

(24) Stephens, P. J.; Devlin, F. J.; Chabalowski, C. F.; Frisch, M. J. *J. Phys. Chem.* **1994**, *98*, 11623–11627.

(25) Grimme, S.; Antony, J.; Ehrlich, S.; Krieg, H. *J. Chem. Phys.* **2010**, *132*, 154104.

(26) Frisch, M. J.; Pople, J. A.; Binkley, J. S. *J. Chem. Phys.* **1984**, *80*, 3265–3269.

(27) Verstraelen, T.; Vandenbrande, S.; Heidar-Zadeh, F.; Vanduyfhuys, L.; Van Speybroeck, V.; Waroquier, M.; Ayers, P. W. *J. Chem. Theory Comput.* **2016**, *12*, 3894–3912.

(28) Verstraelen, T.; Tecmer, P.; Heidar-Zadeh, F.; Boguslawski, K.; Chan, M.; Zhao, Y.; Kim, T. D.; Vandenbrande, S.; Yang, D.; González-Espinoza, C. E.; Fias, S.; Limacher, P. A.; Berrocal, D.; Malek, A.; Ayers, P. W. HORTON 2.0.0. 2015; `http://theochem.github.com/horton/`.

(29) Vanduyfhuys, L.; Vandenbrande, S.; Verstraelen, T.; Schmid, R.; Waroquier, M.; Van Speybroeck, V. *J. Comput. Chem.* **2015**, *36*, 1015–1027.

(30) Vanduyfhuys, L.; Vandenbrande, S.; Wieme, J.; Waroquier, M.; Verstraelen, T.; Van Speybroeck, V. *J. Comput. Chem.* **2018**, *39*, 999–1011.

(31) Chen, J.; Martínez, T. J. *Chem. Phys. Lett.* **2007**, *438*, 315–320.

(32) Bush, B. L.; Bayly, C. I.; Halgren, T. A. *J. Comput. Chem.* **1999**, *20*, 1495–1516.

(33) Allinger, N. L.; Yuh, Y. H.; Lii, J.-H. *J. Am. Chem. Soc.* **1989**, *111*, 8551–8566.

(34) Ghysels, A.; Verstraelen, T.; Hemelsoet, K.; Waroquier, M.; Van Speybroeck, V. *J. Chem. Inf. Model.* **2010**, *50*, 1736–1750.

(35) Cui, Q.; Bahar, I. *Normal Mode Analysis: Theory and Applications to Biological and Chemical Systems*; CRC Press, 2005.

(36) Ghosh, P.; Kim, K. C.; Snurr, R. Q. *J. Phys. Chem. C* **2014**, *118*, 1102–1110.

(37) Rappe, A. K.; Casewit, C. J.; Colwell, K. S.; Goddard, W. A.; Skiff, W. M. *J. Am. Chem. Soc.* **1992**, *114*, 10024–10035.
